# Multidisciplinary intervention for adverse events associated with ATZ + BEV therapy: a case report

**DOI:** 10.1186/s40780-025-00448-z

**Published:** 2025-05-08

**Authors:** Ko Masaki, Motoyasu Miyzaki, Kota Mashima, Yasutaka Sumi, Kohei Noda, Syohei Ueno, Takashi Tanaka, Nobutaka Takahashi, Susumu Kaneshige, Hidetoshi Kamimura

**Affiliations:** 1 Department of Pharmacy, Fukuoka University Hospital, 7-45-1 Nanakuma, Jounan, Fukuoka, 814-0180 Japan; 2 Department of Pharmacy, Fukuoka University Chikushi Hospital, 1-1-1 Zokumyouin, Chikushino, Fukuoka Japan; 3 Department of Gastroenterology, Fukuoka University Hospital, 7-45-1 Nanakuma, Jounan, Fukuoka Japan; 4 Department of Neurology, Fukuoka University Hospital, 7-45-1 Nanakuma, Jounan, Fukuoka Japan; 5 Faculty of Pharmacy, Fukuoka University, 8-19-1 Nanakuma, Jounan, Fukuoka Japan

**Keywords:** irAE, Atezolizumab, Hepatocellular carcinoma, Peripheral neuropathy

## Abstract

**Background:**

Atezolizumab (ATZ) plus bevacizumab (BEV) combination therapy has recently been approved for the treatment of unresectable hepatocellular carcinoma. However, immune-related adverse events (irAEs), including peripheral neuropathy, have also been reported. This case report describes a multidisciplinary intervention for a patient who developed peripheral neuropathy as an irAE following ATZ+BEV combination therapy.

**Case presentation:**

The patient was a 60-year-old man with a history of hypertension. ATZ + BEV combination therapy was initiated for unresectable hepatocellular carcinoma on day 0. On day 6, he experienced a grade 2 hypertensive episode with a systolic blood pressure of 160 mmHg, despite being on amlodipine (5 mg) and azilsartan (20 mg). Based on the pharmacist’s recommendations, the amlodipine dose was increased to 10 mg. However, as hypertension persisted, an additional 20 mg of azilsartan was prescribed, ultimately stabilizing the patient’s blood pressure to approximately 110/60 mmHg. On day 23, the patient reported numbness in his extremities, which was later diagnosed as grade 3 peripheral neuropathy. Notably, data from the IMbrave150 trial indicated that the of peripheral neuropathy as an irAE was 1.5%. This prompted a consultation with a neurologist. Prednisolone (40 mg/day) was initiated on day 26, followed by steroid pulse therapy with methylprednisolone (1000 mg/day for three days) starting on day 37. Despite these interventions, the symptoms did not improve. Rehabilitation therapy was commenced on day 42 after steroid tapering. On day 48, the patient underwent a five-day course of high-dose intravenous immunoglobulin therapy, which also failed to yield improvement. Rehabilitation efforts subsequently shifted to enhancing activities of daily living. Initially, the patient required assistance to stand and faced significant difficulty walking. With consistent strength and mobility training, the patient progressed to walking with crutches and demonstrated increased walking distance.

**Conclusions:**

The pathophysiology of irAE-induced peripheral neuropathy associated with immune checkpoint inhibitors remains poorly understood. This case underscores the challenges of managing irAE-related neuropathy, which may exhibit limited responsiveness to conventional treatments. Early detection, timely intervention, and multidisciplinary approaches are crucial for optimizing patient outcomes and mitigating the impact of severe side effects.

## Background

In recent years, immune checkpoint inhibitors (ICIs) have demonstrated remarkable efficacy in the treatment of various solid and hematologic malignancies. In Japan, the anti-programmed cell death ligand 1 (PD-L1) antibody, atezolizumab (ATZ), a representative ICI, was approved in September 2020 for combination therapy with the anti-vascular endothelial growth factor (VEGF) humanized monoclonal antibody bevacizumab (BEV) for hepatocellular carcinoma. ICIs have shown significant therapeutic benefits when used in combination with cytotoxic agents, small-molecule drugs, anti-angiogenic agents, or radiotherapy [1–3]. ICIs exert their effects by inhibiting immune checkpoint molecules that suppress immune responses, thereby activating T-cells [4]. Unlike conventional cytotoxic drugs or molecular targeted agents, the mechanisms of action and side effect profiles of ICIs are distinct. A notable feature of ICIs is their ability to induce immune-related adverse events (irAEs), which result from a breakdown in immune tolerance, leading to autoimmune-like symptoms [5]. Although irAEs involving the neurological, muscular, and articular systems are relatively rare, peripheral neuropathies appear to occur more frequently than central nervous system toxicities [6, 7].

This report presents a case of peripheral neuropathy as an irAE following ATZ and BEV combination therapy for hepatocellular carcinoma. Despite pharmacological interventions including corticosteroids, the patient exhibited a poor response to treatment. Peripheral neuropathy associated with ICIs is a rare condition with limited treatment options and reports on its management are scarce. We herein describe a case involving a multidisciplinary treatment approach, emphasizing the challenges in managing this rare and poorly responsive condition.

## Case presentation

Patient: A male in his 60s.

Main Complaint: Numbness from the wrists to the fingertips and from the ankles to the toes.

The patient’s medical history included human T-cell leukemia virus type 1 (HTLV-1) infection, hepatitis B virus (HBV) infection (HBs-positive), and hypertension.

Medication history: Tenofovir (25 mg/day), ursodeoxycholic acid (300 mg/day), L-isoleucine, L-leucine, and L-valine combination granules, amlodipine (5 mg), azilsartan (20 mg).

History of current illness: On July 26, 20XX, the patient presented with fatigue, and blood tests revealed an elevated inflammatory response. Antibacterial therapy with lascufloxacin was then initiated. Despite the treatment, the fever persisted. Contrast-enhanced abdominal computed tomography (CT) revealed a large mass in the right lobe of the liver. Subsequently, the patient was diagnosed with hepatocellular carcinoma (T2 N0M0, cStage II, BCLC A) (Table 1).

**Table 1:** Laboratory data

Day0					
WBC	7800	/μL	AST	31	U/L
RBC	405	×10^3^/μL	ALT	60	U/L
Hb	10.7	g/dL	ALP	197	U/L
Ht	33.5	%	γ-GTP	77	U/L
Plt	501	×10^3^/μL	T-Bil	0.4	mg/dL
Lympho	33.5	%	LDH	220	U/L
AFP	1	ng/mL	Alb	2.8	g/dL
PIVKA-II	923	mAU/mL	Na	139	mEq/L
FT4	1.24	ng/dL	K	4.3	mEq/L
FT3	3.14	pg/mL	Cl	105	mEq/L
TSH	2.34	mIU/L	BUN	25	mg/dL
Cortisol	15.4	μg/dL	Cre	1.13	mg/dL
ACTH	19.7	pg/mL	eGFR	49.5	ml/min
Day22					
WBC	6800	/μL	AST	43	U/L
RBC	340	×10^3^/μL	ALT	72	U/L
Hb	9.1	g/dL	ALP	144	U/L
Ht	28.4	%	γ-GTP	83	U/L
Plt	473	×10^3^/μL	T-Bil	0.5	mg/dL
Lympho	24.5	%	LDH	213	U/L
Cortisol	15.5	μg/dL	Alb	2.5	g/dL
ACTH	26.1	pg/mL	Na	131	mEq/L
			K	4.6	mEq/L
			Cl	100	mEq/L
			BUN	16	mg/dL
			Cre	0.81	mg/dL
			eGFR	71.3	ml/min

*ACTH * adrenocorticotropic hormone, *AFP* alpha-hetoprotein, *Alb* albumin, *ALP* alkaline phosphatase, *ALT* alanine aminotransferase, *AST* aspartate aminotransferase, *BUN* blood urea nitrogen**,** *CCr* creatinine clearance, *Cl* chloride, *CRP* C-reactive protein, *FT* free thyroxine, *GFR* estimated glomerular filtration rate, *Hb* hemoglobin, *Ht* hematocrit, *K* potassium, *lympho* lymphocyte, *Na* sodium, *PLT* platelet, *RBC* red blood cell, *T-Bil* total bilirubin, *Tg* thyroglobulin, *TSH* thyroid-stimulating hormone, *WBC* white blood cell

### Primary drug treatment

On August 16, lenvatinib treatment at a dose of 8 mg/day was initiated. ATZ plus BEV combination therapy is generally recommended as first-line drug therapy for hepatocellular carcinoma [8]. However, a case of rapid progression (acute transformation) of adult T-cell leukemia/lymphoma (ATL) was reported following anti-programmed cell death 1 (PD-1) antibody treatment for HTLV-1 carriers with low-grade ATL (chronic type) [9]. This raised concerns regarding the use of immune checkpoint inhibitors in such patients. Given the consideration of conversion surgery and the potential for bleeding complications associated with BEV, the patient was started on lenvatinib (8 mg/day) for 5 consecutive days, followed by a 2-day rest period.

### Second-line drug therapy and progression to the diagnosis of the irAE

Following first-line drug therapy, CT performed on October 30 revealed no reduction in tumor size. Consequently, second-line drug therapy with ATZ plus BEV was initiated on November 7. On November 18, the patient underwent a blood test and computed tomography (CT) scan due to fever and elevated inflammatory markers. The fever was initially attributed to tumor-related activity, and naproxen (300 mg/day) was prescribed. On November 27, the patient experienced grade 2 epistaxis according to the Common Terminology Criteria for Adverse Events (CTCAE) v5.0. The patient was referred to the otolaryngology department for cauterization and hemostasis. On November 29, the patient reported numbness from the wrists to the fingertips and from the ankle to the toes, which persisted for 4–5 days. The numbness progressed, resulting in difficulty in extracting oral medication from the press through the package (PTP) sheet. The condition was classified as grade 3 peripheral neuropathy due to its significant impact on daily activities.

The pharmacist notified the attending physician of the potential irAEs, and further evaluations were initiated. Considering the prior epistaxis, a simple CT scan of the head was performed to rule out intracranial hemorrhage. By December 1, the patient’s symptoms had worsened, progressing to gait disturbance. Prednisolone (40 mg/day) was administered for its neuroprotective effect. A neurological consultation revealed symmetrical muscle weakness in the lower limbs and diminished tendon reflexes. Magnetic resonance imaging (MRI) of the head showed no evidence of meningeal dissemination or metastatic lesions. A cerebrospinal fluid (CSF) analysis revealed the presence of HTLV-1 antibodies, an increased cell count of 13/µL, and elevated protein level (71 mg/dL). These findings suggest the possibility of HTLV-1-associated myelopathy (HAM). Table 2 shows the results of the CSF analysis.

**Table 2:** Cerebrospinal fluid analysis

Day25		
Cells count	13	/μL
Mono	10	%
Total protein(CSF)	71	mg/dL
Immunoglobulin G(blood)	1827	mg/dL
HTLV1 anti body	+	
Day43		
Alb(blood)	2.7	g/dL
Total protein (CSF)	49	mg/dL
A/G	1.62	
Albumin	61.9	%
Immunoglobulin G(blood)	1323	mg/dL
Immunoglobulin G (CSF)	6.4	mg/dL
IgG index	0.44	

*Alb* Albumin, *CSF* Cerebrospinal fluid analysis, *Mono* Monocyte

However, the absence of paraplegia, atypical lymphocytosis, or elevated soluble interleukin-2 receptor (sIL-2R) levels ruled out ATL. Peripheral nerve conduction studies demonstrated sensory deficits, predominantly in the lower extremities with no evidence of central nervous system involvement. Table 3 shows the sIL-2R levels, and Table 4 shows the results of the peripheral nerve conduction studies. Given that the symptoms emerged following the initiation of ATZ treatment, the patient was diagnosed with immune-related peripheral neuropathy secondary to ICIs.

**Table 3:** Laboratory data

	Day14	Day21	Day28	
sIL-2R	4607	2967	1841	U/mL

*sIL-2R* Soluble interleukin-2 receptor

**Table 4:** Nerve conduction studies

	CMAP amplitude during proximal stimulation (mV)	CMAP amplitude during distal stimulation (mV)	MCV (m/s)	F-Latency (ms)
Median nerve				
Right upper extremity	9.3	9.9	49.8	24.8
Left upper extremity	5.8	6.4	47.6	29.7
Tibial nerve				
Right lower extremity	4.7	5.9	34.0	64.9
Left lower extremity	6.6	6.5	34.9	36.2

*CMAP* Compound muscle action potential, *MCV* motor nerve conduction velocity

### Treatment after the diagnosis of peripheral neuropathy as an irAE

**Fig. 1:**
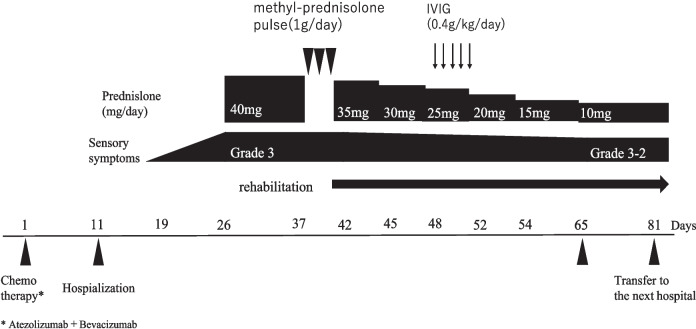
Clinical course

Following the diagnosis of peripheral neuropathy as an irAE, prednisolone (40 mg/day) was administered until December 13. As the symptoms showed no improvement, steroid pulse therapy with methylprednisolone (1000 mg/day) was initiated for three consecutive days. Despite the intervention, the patient’s symptoms persisted. Consequently, prednisolone was gradually tapered, and intravenous immunoglobulin (IVIG) therapy was administered over a 5-day period starting on December 25, in accordance with the cancer immunotherapy guidelines [1]. Given the lack of symptomatic improvement after IVIG therapy, further immunosuppressive treatments were not pursued. Instead, the patient was advised to continue rehabilitation with the goal of enhancing activities of daily living (ADLs). Figure 1 shows the progression of the patient’s symptoms and the subsequent therapeutic interventions.

Sixty-year-old man who received atezolizumab for unresectable hepatocellular carcinoma. The unusual clinical course including short nadir period indicated the diagnosis of acute inflammatory demyelinating polyneuropathy caused by atezolizumab.

### Multidisciplinary cooperation

#### Pharmacological intervention

**Fig. 2:**
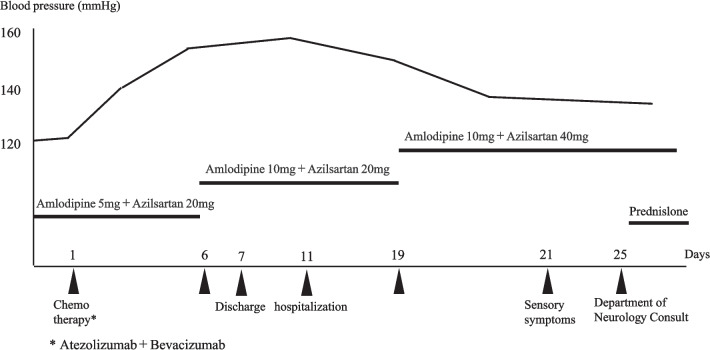
Pharmacological intervention

On November 7 (day 0), the patient was started on ATZ plus BEV combination therapy as second-line drug therapy. By day 6, the patient had a systolic blood pressure of 160 mmHg, which was classified as grade 2 hypertension. The patient’s initial antihypertensive regimen included amlodipine (5 mg) and azilsartan (20 mg). In response to the elevated blood pressure, the pharmacist recommended an additional 5 mg of amlodipine. However, the systolic blood pressure remained at 150 mmHg, prompting further recommendation to add another 20 mg of azilsartan. Subsequently, the patient’s blood pressure improved and stabilized at approximately 110/60 mm Hg. Figure 2 shows the trends in systolic blood pressure and the corresponding pharmacological interventions.

This patient was treated with atezolizumab for unresectable hepatocellular carcinoma and developed grade 2 hypertension on Day 6, which resulted in a drug adjustment, and the transition of the antihypertensive drug.

On day 23, the pharmacist conducted a follow-up interview during which the patient reported numbness in the extremities. A review of newly administered drugs, including amlodipine, azilsartan, naproxen, and ATZ plus BEV, was performed to assess potential adverse drug reactions (ADRs). Reports of peripheral neuropathy associated with amlodipine (<0.1%) and naproxen (<0.1%) have been published. In contrast, ATZ plus BEV combination therapy resulted in a higher incidence of neuropathy (1.5% for all grades) in IMbrave150 [10]. The median (range) time to the onset of neuropathy in the study was 65.5 days (26–145 days), which overlapped with the patient’s clinical course. Although the number of reported cases was extremely limited, the incidence appeared relatively high compared to peripheral neuropathy caused by other agents. The pharmacist raised the possibility of irAE peripheral neuropathy and recommended consultation with the Department of Neurology.

#### Rehabilitation intervention

**Fig. 3:**
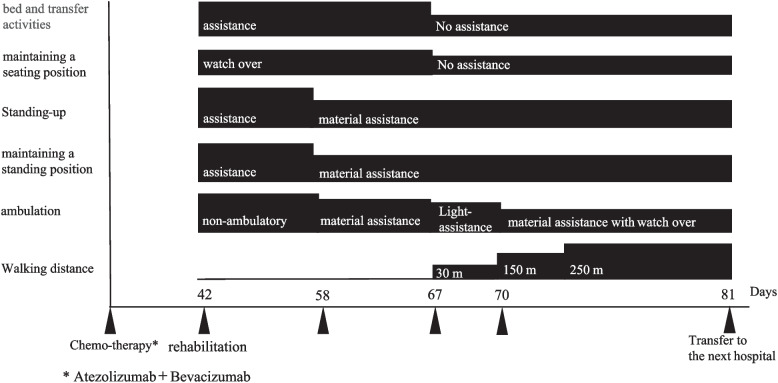
Rehabilitation course

Peripheral neuropathy induced by ATZ plus BEV combination therapy progressed to gait disturbance beginning on day 23. Despite systemic steroid therapy and high-dose IVIG, the patient’s symptoms showed no significant improvement. Figure 3 shows the changes in patients’ ADLs following rehabilitation interventions.

The patient developed peripheral neuropathy, and since pharmaceutical intervention was not sufficient to improve the condition, the goal was to expand ADLs through rehabilitation intervention. The following figure shows the change in ADLs due to rehabilitation.

Rehabilitation was commenced on day 42. Initially, the patient required assistance in standing and could not walk independently. Early rehabilitation efforts have focused on strength and movement training. By day 58, the patient was able to stand with physical support such as a handrail. Subsequently, gait training was initiated using parallel bars and crutch training. By day 67, the patient had achieved independent ambulation using crutches. Continued lower limb strength training and gait training further increased walking distance. Although significant improvements in ADLs were observed, the patient retained grade 2 neuropathic symptoms. With successful rehabilitation, five months after the onset of peripheral neuropathy, lenvatinib was restarted for the treatment of hepatocellular carcinoma.

## Discussion and conclusions

CD8+ T cells play a central role in antigen recognition and tumor cell attacks in cancer immunotherapy. Their activation requires a primary signal mediated by the major histocompatibility complex (MHC) and a secondary co-stimulatory signal via molecules, such as CD28 [11, 12]. However, immune checkpoint molecules, including cytotoxic T-lymphocyte-associated protein 4 (CTLA-4) and PD-1/PD-L1, act as regulators that suppress immune hyperactivation and maintain immune homeostasis. These molecules are also implicated in autoimmune diseases [13], and antibodies targeting them–ICIs–have been widely adopted in oncology. Although they are effective, ICIs can cause irAEs that mimic autoimmune and inflammatory diseases [5].

ATZ plus BEV combination therapy is the first-line drug therapy for unresectable advanced hepatocellular, according to international guidelines [8]. However, irAEs, including peripheral neuropathy, remain concerning. Between 2012 and 2024, WHO VigiBase recorded 149 cases of peripheral neuropathy associated with ATZ [14]. In the IMbrave150 study, the incidence of neuropathy in patients treated with ATZ and BEV was 1.5% in all grades [10]. Although this incidence is relatively low, severe cases have been reported, necessitating early intervention [15].

In this case, the patient developed grade 3 peripheral neuropathy 21 days after starting the ATZ plus BEV combination therapy. The symptoms included muscle weakness predominantly in the left upper and lower extremities, numbness from the shoulder joint to the periphery, sock-type hyperalgesia and numbness in the lower extremities, decreased tendon reflexes, impaired deep sensation, and gait disturbance. Despite systemic steroid administration, steroid pulse therapy, and high-dose IVIG treatment, the patient’s symptoms did not improve. Diagnostic evaluations, including contrast-enhanced MRI and the absence of malignant findings on CSF cytology, revealed no evidence of meningeal dissemination, metastasis, or central nervous system involvement. However, mild compound motor action potential (CMAP) depression and sensory neuropathy suggest neuronopathy. This case exhibited peripheral neuropathy with a predominant impairment of deep sensation, differential diagnoses included paraneoplastic syndrome, Sjögren’s syndrome, prior chemotherapy with platinum agents, and excessive vitamin B6 intake. Given the temporal relationship between the administration of ATZ and the onset of symptoms, irAE peripheral neuropathy was deemed to be the most likely diagnosis. Paraneoplastic syndrome was judged to be negative because the Immunoglobulin G (IgG) index was not elevated at 0.44, the oligoclonal band was negative, and the patient developed symptoms immediately after receiving ICIs. Sjogren’s syndrome was ruled out because of negative results for anti-SS-A and anti-SS-B antibodies and the absence of dry eye and dry mouth symptoms. The patient had no history of chemotherapy with platinum agents or of excessive vitamin B6 intake.

The patient’s status as an HTLV-1 carrier complicates the clinical picture. There have been previous reports of ATL due to reactivation after administration of the PD-1 antibody [9]. Since there was no increase in abnormal lymphocytes, it was determined that this was not an acute exacerbation of ATL, and the patient was followed up. However, a CSF analysis showed elevated HTLV-1 antibody titers, cell counts, and protein levels, raising the possibility of HAM. However, the absence of paraplegia and normal sIL-2R levels make acute ATL unlikely. Differentiating between HAM and irAE-related neuropathy remains challenging because clear diagnostic criteria are lacking.

Yamanaka et al. reported a case of acute demyelinating peripheral neuropathy resembling Guillain-Barré syndrome(GBS) after ATZ administration [16]. Although the detailed pathogenesis of peripheral neuropathy as an irAE caused by ICIs has not been clarified, it is believed to be caused by a different mechanism from the usual GBS [17], since regulatory T cells are said to be the primary pathological agents based on their mechanism of action. According to cancer immunotherapy guidelines [1], irAE peripheral neuropathies include rapidly progressive GBS and chronic inflammatory demyelinating polyradiculoneuropathy, which are appropriately grouped together as polyradiculoneuropathy [18], and most of these symptoms develop within 2 months of the start of ICI administration. Furthermore, Duvey et al. reported that the early use of steroids for irAE peripheral neuropathy results in a favorable neurologic prognosis [7]. Additionally, in a case report by Takahashi et al., IVIG administration was associated with improvements in activities of daily living (ADL). However, in the present case, neither steroid therapy nor high-dose IVIG produced any improvement [15]. In GBS, CSF typically exhibits albuminocytological dissociation; however, this case showed a mild increase in the CSF cell count. Additionally, unlike classical GBS, immune checkpoint inhibitor-induced GBS can present with a chronic course resembling chronic inflammatory demyelinating polyneuropathy (CIDP) [19]. Although the clinical course of this case was consistent with such a presentation, the relatively preserved tibial nerve F-waves on nerve conduction studies and the poor response to steroids were considered atypical for ICI-induced GBS. While ganglioside antibodies in the serum were not measured, the absence of significant albuminocytological dissociation in the CSF, nerve conduction study findings, and limited response to both steroids and intravenous immunoglobulin (IVIG) led us to rule out ICI-induced GBS. In this case, despite the administration of steroid pulse therapy and high-dose IVIG, the sensory disturbances did not improve, leading to the conclusion that the effects of these treatments were limited. Therefore, the final treatment plan focused on improving ADL through continued rehabilitation. Subsequently, with continued rehabilitation, walking improved to a point where it became possible with the use of crutches. However, it is difficult to determine whether the improvement was due to pharmacologic treatment, rehabilitation, or the natural course of recovery. Since the use of ICIs for hepatocellular carcinoma has only recently been approved, the accumulation of cases is awaited in the future.

The approval of the CTLA-4 antibody tremelimumab in combination with the PD-L1 antibody durvalumab for unresectable hepatocellular carcinoma in 2023 expands the treatment options. However, a retrospective observational study indicated that CTLA-4 antibodies carry a higher risk of GBS than PD-1 and PD-L1 antibodies, particularly in combination regimens [20]. Peripheral neuropathies as irAEs induced by ICIs are often reported to involve primarily sensory disturbances; however, their clinical presentations are diverse, and motor neuropathies can also occur. In contrast, peripheral neuropathy caused by taxane- or platinum-based chemotherapies typically presents as glove-and-stocking-type numbness and is generally not associated with objective sensory deficits [1]. Severe cases, such as this one, underscore the importance of early recognition and multidisciplinary management of irAEs.

With the expanding use of ICIs across various malignancies, the continued accumulation of clinical data, including case reports—is essential for optimizing treatment strategies and improving patient outcomes. In this case, rapid multidisciplinary collaboration—beginning with the pharmacist’s early detection of irAE signs during adverse‐event monitoring and reporting to the attending physician, followed by prompt neurologic consultation and the rehabilitation team’s assessment of ADL decline and initiation of early rehabilitation—facilitated the patient’s swift recovery. Going forward, it will be necessary to further systematize information sharing in routine practice and to establish regular multidisciplinary conference forums to ensure the timely detection and effective management of severe irAEs.

## Data Availability

No datasets were generated or analysed during the current study.

## Abbreviations

ADLs: Activities of Daily Livings
ADRs: Adverse drug reactions
ATL: T-cell leukemia/lymphoma
ATZ: Atezolizumab
BEV: Bevacizumab
CIDP: Chronic inflammatory demyelinating polyneuropathy
CMAP: Compound motor action potential
CT: Computed tomography
CTCAE: Common Terminology Criteria for Adverse Events
CTLA-4: Cytotoxic T-lymphocyte-associated protein 4
CSF: Cerebrospinal fluid
GBS: Guillain-Barré syndrome
HAM: HTLV-1 associated myelopathy
HBV: Hepatitis B Virus
HTLV-1: Human T-cell leukemia virus type 1
ICIs: Immune checkpoint inhibitors
IgG: Immunoglobulin G
irAE: Immune-related adverse event
IVIG: Intravenous immunoglobulin
MHC: Major histocompatibility complex
MRI: Magnetic resonance imaging
PD-L1: Programmed cell Death Ligand 1
PD-1: Programmed cell death 1
PTP: Press through package
sIL-2R: Soluble interleukin-2 receptor
VEGF: Vascular endothelial growth factor
WHO: World Health Organization
